# Acute Ileal Diverticulitis: Computed Tomography and Ultrasound Findings

**DOI:** 10.3390/diagnostics13081408

**Published:** 2023-04-13

**Authors:** Lyo Min Kwon, Kwanseop Lee, Min-Jeong Kim, In Jae Lee, Gab Chul Kim

**Affiliations:** 1 Department of Radiology, Hallym University Sacred Heart Hospital, Hallym University College of Medicine, Anyang 14068, Republic of Korea; 2 Department of Radiology, Kyungpook National University Medical Center, Daegu 41404, Republic of Korea

**Keywords:** diverticulitis, ileum, tomography, X-ray computed, ultrasonography, perforation

## Abstract

Background: Acute ileal diverticulitis is a rare disease mimicking acute appendicitis. Inaccurate diagnosis with a low prevalence and nonspecific symptoms leads to delayed or improper management. Methods: This retrospective study aimed to investigate the characteristic sonographic (US) and computed tomography (CT) findings with clinical features in seventeen patients with acute ileal diverticulitis diagnosed between March 2002 and August 2017. Results: The most common symptom was abdominal pain (82.3%, 14/17) localized to the right lower quadrant (RLQ) in 14 patients. The characteristic CT findings of acute ileal diverticulitis were ileal wall thickening (100%, 17/17), identification of inflamed diverticulum at the mesenteric side (94.1%, 16/17), and surrounding mesenteric fat infiltration (100%, 17/17). The typical US findings were outpouching diverticular sac connecting to the ileum (100%, 17/17), peridiverticular inflamed fat (100%, 17/17), ileal wall thickening with preserved layering pattern (94.1%, 16/17), and increased color flow to the diverticulum and surrounding inflamed fat on color Doppler imaging (100%, 17/17). The perforation group had a significantly longer hospital stay than non-perforation group (*p* = 0.002). In conclusion, acute ileal diverticulitis has characteristic CT and US findings that allow radiologists to accurately diagnose the disease.

## 1. Introduction

Acute ileal diverticulitis is a very rare disease that often presents as acute right lower quadrant pain in elderly patients, and it is usually misdiagnosed as acute appendicitis [1,2,3,4,5,6,7]. With a low prevalence and nonspecific symptoms, an accurate preoperative diagnosis was seldom made, which resulted in a high mortality rate of 25–50% [8,9,10,11]. Non-Meckel ileal diverticula are acquired false diverticula, composed of mucosa, submucosa, and serosa. Meckel diverticula, on the other hand, are congenital true diverticula containing all four layers of the intestinal wall, including the muscular layer. Non-Meckel ileal diverticula are usually located along the mesenteric border and can be small, thin-walled, and fragile. Before the advent of computed tomography (CT), ileal diverticula and complications have sometimes been diagnosed using small bowel barium studies or small bowel enteroclysis [12,13,14,15]. Recent developments in CT techniques have enabled radiologists to diagnose small bowel diverticulitis [4,11,16,17,18,19,20,21,22]. Acute ileal diverticulitis may be successfully treated with non-operative management, even in cases of perforated diverticulitis [3,14,19,23,24,25]. Therefore, the accurate diagnosis of acute ileal diverticulitis is very important for proper management.

Ultrasonography (US) is widely used as an initial screening modality for acute abdominal pain due to its noninvasiveness and easy accessibility [26,27,28]. Recent technological advances in US machines have led to dramatic improvements in contrast, spatial, and temporal resolutions for bowel imaging [26,27,28,29]. Ileal diverticula are commonly seen near the ileocecal valve, possibly due to increased intraluminal pressure and reversion of the blood vessels to an arcade system with larger vasa recta [30]. Accordingly, US would be a very useful tool for the evaluation of acute ileal diverticulitis usually seen in the terminal ileum. However, to our knowledge, there is a paucity of literature on US findings of acute ileal diverticulitis in English-language publications [19,20,21].

Therefore, the purpose of this study was to investigate the characteristic US findings with color Doppler imaging (CDI), CT, and clinical findings for acute ileal diverticulitis.

## 2. Materials and Methods

### 2.1. Enrolled Patients

This retrospective study was approved by an ethical committee and the requirement for informed consent was waived. We searched the radiology department databases for cases of ileal diverticulitis diagnosed between March 2002 and August 2017. First, we selected all patients with US or CT reports including the terms “diverticulitis” and “ileum” or “ileal”. Thirty-one patients were identified in this step. Second, we reviewed all the detailed clinical and radiologic data and the available surgical and pathologic reports. Eight patients were excluded with the diagnoses of right colonic diverticulitis (*n* = 3), Crohn’s disease (*n* = 1), tuberculosis (*n* = 1), stump appendicitis after previous appendectomy (*n* = 1), appendicitis located in left lower quadrant (*n* = 1), and appendiceal diverticulitis (*n* = 1). Third, we excluded six patients who did not undergo CT (*n* = 1) or US examination (*n* = 5), because CT or US alone without surgical confirmation is an imperfect reference standard for the diagnosis of acute ileal diverticulitis. Finally, our study included a total of 17 patients with a confirmed diagnosis of acute ileal diverticulitis based on clinical features, CT, and US examinations.

The medical records of the enrolled patients were reviewed by one investigator (LMK). Clinical data on patient age, sex, clinical presentation, body temperature on the day of admission, laboratory findings including white blood cell count, C-reactive protein (CRP) level, clinical provisional impression, treatment, length of hospital stay, and patient outcome were obtained from hospital medical records. The length of hospital stay was defined as the duration from hospital admission to discharge.

### 2.2. Imaging Techniques

All CT examinations were obtained using one of several CT scanners: a 16-slice multi-detector CT (MDCT) scanner (Mx 8000 IDT, Philips Medical Systems, Best, The Netherlands), a 64-slice MDCT scanner (Brilliance 64, Philips Healthcare, Cleveland, OH, USA), or two 128-slice MDCT scanners (SOMATOM Definition Edge or SOMATOM Definition Flash; Siemens Healthineers, Forchheim, Germany). Our CT protocol included unenhanced CT scans and enhanced CT scans with a 70 s delay after the administration of 120 mL of an intravenous contrast medium at a rate of 3 mL/s. The CT scanning parameters were as follows: for 16-detector rows, beam collimation of 1.5 mm × 16, pitch of 1.2, kVp/effective mA of 120/200–300, slice thickness of 5 mm, axial and coronal reconstruction interval of 5 mm; for 64-detector rows, beam collimation of 0.625 mm × 64, pitch of 0.891, kVp/effective mA of 120/240, slice thickness of 5 mm, axial and coronal reconstruction interval of 5 mm; for 128-detector rows, beam collimation of 0.625 mm × 128, gantry rotation time of 0.5 s, kVp/effective mA of 120/140–200, slice thickness of 5 mm, axial and coronal reconstruction interval of 5 mm. 

All US examinations were performed using one of three ultrasound units: IU-22 (Philips Medical System, Bothell, WA, USA) with a 2- to 5-MHz convex array transducer and a 5- to 8-MHz curved transducer; HDI 5000 (Advanced Technology Laboratory, Bothell, WA, USA) with a 2- to 5-MHz convex array transducer and a 5- to 8-MHz curved transducer; Logic E9 (General Electric, Milwaukee, WI, USA) with a 1- to 6-MHz abdominal sector transducer and 9-MHz linear probe. CDI was also performed in all patients. Trainees and attending radiologists were involved in the US scanning. Final confirmation was performed by one abdominal radiologist with 30 years of experience (K.L.).

### 2.3. Image Analyses

All CT images were retrospectively reviewed by two radiologists (M.-J.K. and G.C.K.) with 15 and 18 years of clinical experience by consensus. The following CT features were evaluated: number of ileal diverticulum (single or multiple); location of ileal diverticulum (mesenteric or antimesenteric); inflamed diverticulum—identification, size, presence or absence of internal fecalith; mesenteric fat infiltration (mild, peridiverticular infiltration; moderate, confined to mesentery; severe, beyond the mesentery); ileal wall thickening; presence or absence of perforation—abscess, extraluminal fluid with air, focal defect in the diverticular sac; venous gas; venous thrombosis; small bowel ileus; normal appendix; other diverticula in the small or large bowels. Diverticulum was defined as an outpouching sac connecting to the bowel. Inflamed diverticulum was defined as a diverticulum with an enhanced or thickened wall at the center of mesenteric inflammation. The size of the inflamed diverticulum was considered the maximal diameter of the inflamed diverticular sac. The presence of fecalith was defined as radiodense material within the inflamed diverticulum. Perforation was considered to be one or more of the following three CT findings: abscess, extraluminal fluid with air, or focal defect in the diverticular sac.

All US images were also evaluated as follows: number of ileal diverticulum (single or multiple); inflamed diverticulum—identification, size, echogenicity (homogeneous hypoechoic sac; hypoechoic sac with internal strong echo; central hyperechoic fecalith with peripheral hypoechoic rim); peridiverticular inflamed fat; ileal wall thickening; presence or absence of perforation—abscess, extraluminal air bubble; normal appendix. We also evaluated the presence or absence of increased color flow of the diverticulum and surrounding fat on CDI. Inflamed diverticulum was defined as an outpouching sac connecting to the bowel at the center of mesenteric inflammation. The size and echogenicity of the inflamed diverticulum were measured at the largest inflamed diverticulum, if there were multiple ones. Peridiverticular inflamed fat was considered non-compressible hyperechoic fat around the diverticulum. An extraluminal air bubble presenting as tiny hyperechoic reverberating dots or abscess formation was considered perforation.

The enrolled patients were divided into perforation and non-perforation groups after excluding one patient who was transferred to another hospital. The perforation group included the patients showing perforation on CT or US imaging. The two groups were compared in terms of age, sex, fever, leukocytosis, length of hospital stay, and inflamed diverticular size. 

### 2.4. Statistics

Descriptive statistics were used to express percentages, means, and standard deviations for continuous and categorical data. The Mann–Whitney test and Fisher’s exact test were used to compare the perforation and non-perforation groups. All statistical analyses were performed using SPSS version 24.0 (IBM Corporation, Armonk, NY, USA); *p* < 0.05 was considered to be statistically significant.

## 3. Results

### 3.1. Clinical Features, Treatment, and Outcomes

All 17 patients were diagnosed with acute ileal diverticulitis. There were 11 men and six women with a median age of 59 years (range, 32–78 years). The most common clinical symptom was abdominal pain in 14 patients (82.3%), localized to right lower quadrant (RLQ). Associated symptoms were febrile sense in seven, vomiting in four, chills in four, diarrhea in three, and nausea in two patients. RLQ tenderness was reported in 14 patients (82.3%). Fever (>37.3 °C) was reported in seven (46.7%) of 15 patients who measured body temperature. Leukocytosis (>10,000/mL) was seen in 12 (80%) of 15 patients and CRP was high in all 13 patients (100%) who were measured. The presumptive clinical impression after history taking and physical examination were appendicitis (*n* = 10), stump appendicitis with previous appendectomy history (*n* = 1), diverticulitis (*n* = 2), fever with unknown origin (*n* = 2), cholecystitis (*n* = 1), and peritoneal dialysis-related peritonitis (*n* = 1). All 17 patients underwent both US and CT examinations. The first diagnostic modality was CT in ten patients and US in seven patients. The interval time between the two imaging modalities was within 5 days: same day in 8, 1 day in 5, 2 days in 2, 4 days in 1, and 5 days in 1 patient. Three of the 17 patients underwent barium study and they all revealed single or multiple ileal diverticula along the mesenteric border. Fifteen of the 17 patients received oral or intravenous antibiotics and fully recovered without complications. One patient successfully recovered with conservative treatment without antibiotics. The remaining patient, who was recommended to undergo surgery, was transferred to another hospital and was lost to further follow-up (Table 1).

### 3.2. CT Findings

**Figure 1:**
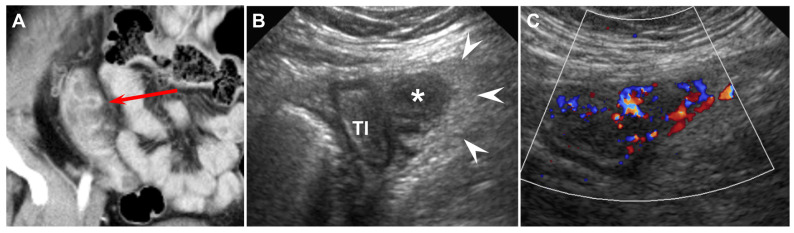
Forty-eight-year-old female (patient #8) with acute ileal diverticulitis. (**A**) Coronal reformatted enhanced CT scan reveals an outpouching diverticular sac (red arrow) connecting to the terminal ileum and mild ileal wall thickening. (**B**) US scan with C8-5 convex transducer demonstrates a homogeneous hypoechoic diverticular sac (*) connecting to the terminal ileum (TI), surrounding hyperechoic inflamed fat (arrowheads), and mild wall thickening of terminal ileum (TI). (**C**) Color Doppler imaging shows increased color flow to the diverticular wall and surrounding inflamed fat.

**Figure 2:**
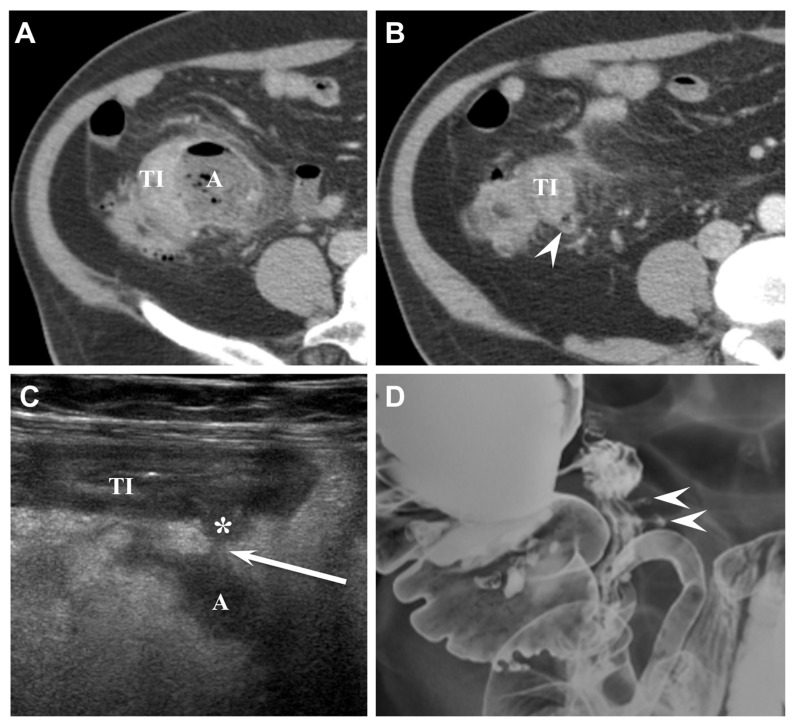
Sixty-seven-year-old male (patient #2) with acute perforated ileal diverticulitis. (**A**,**B**) Axial CT scans show ileal wall thickening (TI) with mesenteric abscess (A) and mesenteric fat infiltration. There is no definite inflamed diverticulum on CT. However, adjacent ileal diverticulum (arrowhead) supports the diagnosis of perforated ileal diverticulitis with abscess formation. (**C**) US scan with linear transducer reveals outpouching inflamed diverticulum (*) protruding from the terminal ileum (TI) and connecting to mesenteric abscess (A) with focal perforation site (arrow). (**D**) Barium study after 1 month shows two diverticula (arrowheads) protruding from the terminal ileum and multiple diverticula from the colon.

**Figure 3:**
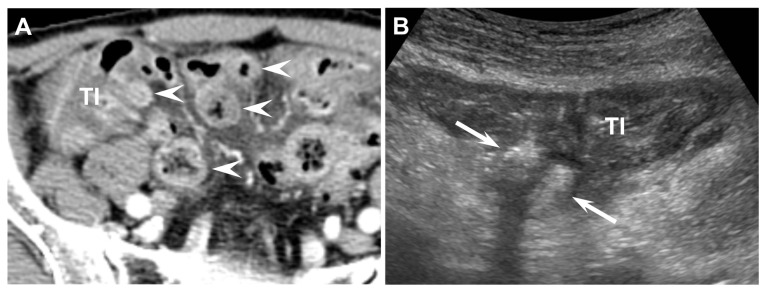
Seventy-eight-year-old male (patient #4) with acute ileal diverticulitis. (**A**) Enhanced axial CT scan shows multiple ileal diverticula (arrowheads) with or without fecalith, ileal wall thickening (TI), and mesenteric fat infiltration. (**B**) US scan with C5-1 convex transducer demonstrates two outpouching diverticular sacs (arrows) with central hyperechoic fecalith, ileal wall thickening (TI), and increased peridiverticular fat echogenicity.

**Figure 4:**
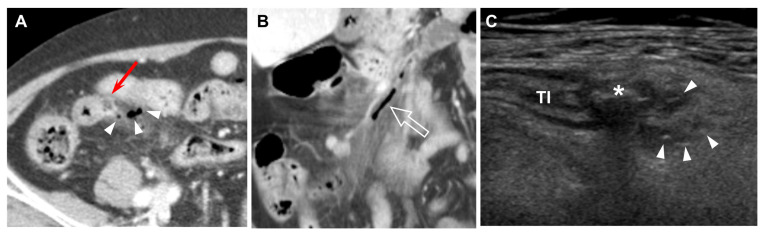
Sixty-three-year-old female (patient #9) with acute perforated ileal diverticulitis. (**A**,**B**) Axial and coronal reformatted CT scans show inflamed diverticulum (red arrow), with extraluminal gas (arrowheads) representing perforation. Mesenteric fat infiltration, terminal ileal wall thickening, and mesenteric venous gas (open arrow) are also seen. (**C**) US scan demonstrates an outpouching inflamed diverticulum with central hyperechoic fecalith (*) connecting to terminal ileum (TI), and tiny hyperechoic reverberating dots (arrowheads) represent extraluminal air bubbles.

**Figure 5:**
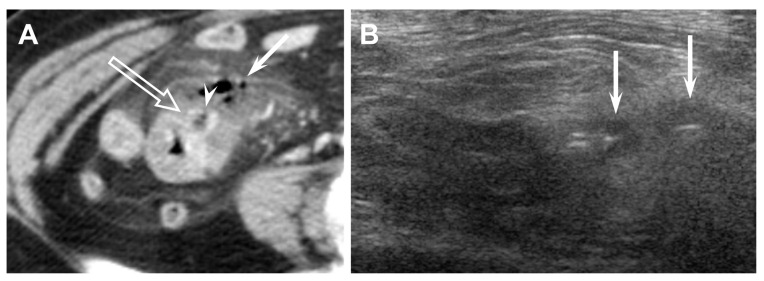
Seventy-year-old female (patient #7) with acute perforated ileal diverticulitis. (**A**) Axial CT scan shows terminal ileal wall thickening, an outpouching inflamed diverticulum (open arrow) with focal wall defect (arrowhead) at the mesenteric side of terminal ileum, extraluminal air with fluid (arrow), and mesenteric fat infiltration. (**B**) US scan with linear transducer also reveals extraluminal air bubbles (arrows) representing perforation.

The CT findings for acute ileal diverticulitis are summarized in Table 2. All 17 patients had ileal diverticulum along the mesenteric border. Inflamed diverticulum was seen in 16 patients (94.1%) (Figure 1). The remaining patient did not have an inflamed diverticulum at the center of ileal wall thickening with abscess; however, adjacent ileal diverticula supported the diagnosis of acute ileal diverticulitis with perforation, and barium study after 1 month demonstrated two ileal diverticula along the mesenteric border (Figure 2). Three patients had radiodense fecalith within the inflamed diverticulum (Figure 3). All 17 patients had mesenteric fat infiltration with varying degrees and ileal wall thickening. Five patients (29.4%) were diagnosed with perforated ileal diverticulitis, which had the following findings: abscess (*n* = 2) (Figure 2), extraluminal fluid with air (*n* = 3) (Figure 4), and/or focal defect in the diverticular sac (*n* = 2) (Figure 5). Mesenteric venous gas (Figure 4) and mesenteric venous thrombosis were seen in two patients (11.8%), respectively. 

### 3.3. US Findings

**Figure 6:**
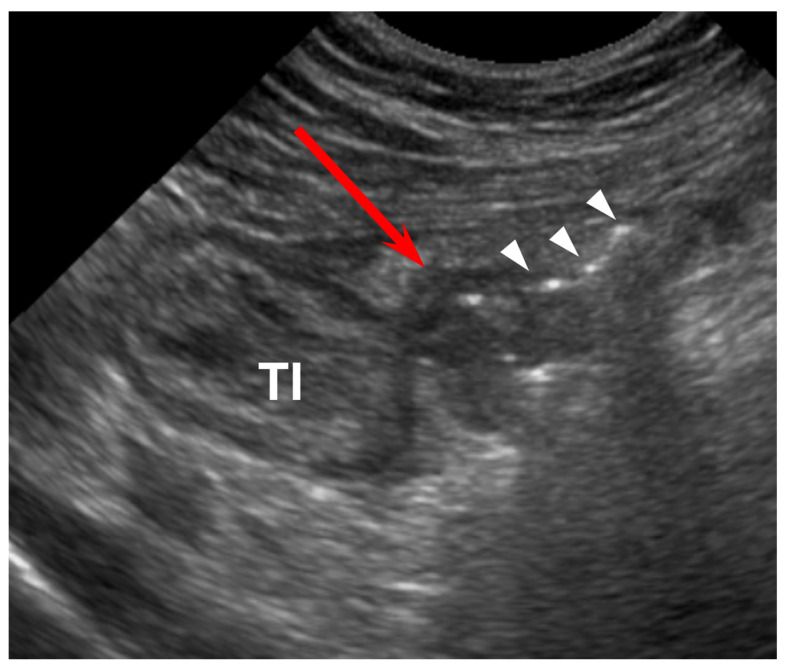
Forty-two-year-old male (patient #5) with acute perforated ileal diverticulitis. Axial US scan reveals ileal wall thickening (TI), an outpouching hypoechoic diverticular sac (red arrow) and linear reverberating strong echoes (arrowheads) in the mesentery, suggesting perforation.

The US findings for acute ileal diverticulitis are summarized in Table 3. All patients showed outpouching inflamed diverticular sac connecting to the ileum, peridiverticular inflamed fat presenting as hyperechoic fat around the diverticulum, and increased color flow to the diverticulum and surrounding inflamed fat on CDI (Figure 1). The inflamed diverticulum exhibited variable echogenicity (Figure 2 and Figure 3). Like CT findings, eight patients had a single diverticulum and nine patients had multiple diverticula. Unlike CT, US examinations diagnosed perforated ileal diverticulitis in seven patients. The findings indicated that five patients had both abscess and extraluminal air bubble (Figure 4 and Figure 5), one patient had only extraluminal air bubble (Figure 6), and one patient had only abscess.

### 3.4. Comparison between Perforation and Non-Perforation Groups

The perforation group consisted of seven patients and the non-perforation group consisted of nine patients. The perforation group had a significantly longer hospital stay than the non-perforation group (*p* = 0.002). The size of the inflamed diverticulum in the perforation group was smaller than that in the non-perforation group (*p* = 0.017). However, there were no significant differences between the two groups in age, sex, fever, and leukocytosis (Table 4).

## 4. Discussion

Non-Meckelian jejunoileal diverticula are rare, acquired false diverticula, with reported incidence rates of 0.5% to 2.3% on small bowel contrast studies [12,31]. Considering that approximately 20% of jejunoileal diverticula occur in the ileum and only 10% of them develop complications, acute ileal diverticulitis is an extremely rare disease despite it being the most common complication of ileal diverticulosis [1,2,3,4,5,6,7,23,24]. Our hospital, which has a total of 836 beds, including 80 beds allocated to surgical wards, collected a total of 17 cases over a 15-year period, further demonstrating the rarity of this condition. Additionally, acute ileal diverticulitis was more common in male patients (male to female ratio, 11:6) and elderly patients, with a median age of 59 years (range, 32–78 years), in this study. Most patients presented with abdominal pain, especially right lower quadrant pain, fever, leukocytosis, and CRP elevation. Therefore, most patients were diagnosed as acute appendicitis initially. These were similar to the result of previous studies [1,2,3,4,5,6,7,10,13,14,15,23]. Preoperative misdiagnosis of this disease results in emergent surgery with or without postoperative complications. With the widespread use of CT and US in the evaluation of patients with acute abdominal pain [3,4,5,6,7,9,11,16,20,21,22,32,33], it has been important to recognize the CT and US findings of acute ileal diverticulitis and to include them in the differential diagnoses because this disease is often not suspected clinically.

Historically, acute ileal diverticulitis has been diagnosed on exploratory laparotomy for other challenging differential diagnoses [1,20]. The technological advances and widespread use of CT have enabled radiologists to play an essential role in diagnosing acute ileal diverticulitis [3,16,18]. The characteristic CT findings of acute ileal diverticulitis in this study included ileal wall thickening with small-sized inflamed diverticulum at the mesenteric side and surrounding mesenteric fat infiltration, which was consistent with CT findings in previous studies [3,4,5,6,7,8,9,10,11,13,16,18,24,30]. Especially, direct visualization of the inflamed diverticulum was the key feature in diagnosing acute ileal diverticulitis. Sixteen of 17 patients had direct visualization of inflamed diverticulum on CT. The remaining patient, who was diagnosed with perforated diverticulitis with abscess, did not show an inflamed diverticulum on CT. However, US demonstrated inflamed diverticulum protruding from the ileum and connecting with the abscess in the patient. We believe that US could play a complementary role if the inflamed diverticulum was not seen on CT.

To our knowledge, there has been no study focused on US findings with CDI of acute ileal diverticulitis, although there have been published isolated case reports with only one case each [20,21]. This study was the largest reported number of acute ileal diverticulitis with US findings. The characteristic US findings were outpouching inflamed diverticular sac connecting to the ileum, hyperechoic peridiverticular inflamed fat, ileal wall thickening with preserved layering structure, and increased color flow to the diverticulum and surrounding inflamed fat on CDI. These US findings are similar to those of a few case reports of acute ileal diverticulitis [20,21] and right colonic diverticulitis [34,35]. Differential diagnoses presenting as acute RLQ pain include appendicitis, cecal diverticulitis, terminal ileitis, and Crohn’s disease. The diagnosis of acute ileal diverticulitis would be easier with the recognition of differentiation points including knowledge of accurate and detailed US anatomy of the ileocecal area, visualization of normal appendix, ileal wall thickening with preserved layering structure, and detection of outpouching inflamed diverticular sac. 

Although guidelines for the management of acute ileal diverticulitis have not been established and previous studies have reported that perforated ileal diverticulitis should be treated with surgical excision, recent studies suggest that acute ileal diverticulitis can be successfully treated with conservative management, even in cases with perforated ileal diverticulitis with or without abscess unless generalized peritonitis was not seen on radiologic examinations [3,24]. Similarly, our study showed that 16 of 17 patients, even those with perforated ileal diverticulitis, successfully recovered after conservative treatment with or without antibiotics. The perforation group had a significantly longer hospital stay than the non-perforation group (*p* = 0.002). The perforation may be a predictive factor for poor clinical outcomes in acute ileal diverticulitis. Interestingly, the diverticular size was smaller in the perforation group than in the non-perforation group (*p* = 0.017), presumably because the inflamed diverticulum collapsed after perforation. 

This study has some limitations. First, this retrospective design could not completely exclude selection bias because only patients who underwent both CT and US examinations were included. We excluded the patients who did not undergo CT or US examination because CT or US alone is an imperfect reference standard for the diagnosis of acute ileal diverticulitis. The mild form of ileal diverticulitis may have been overlooked due to the presentation of mild non-specific abdominal pain, and CT or US examinations may not have been performed. Another source of selection bias is possible misdiagnosis as another condition because of mistakes in image interpretation. Second, we had a small sample of patients. However, acute ileal diverticulitis is extremely rare, and only limited case reports are available. Unlike previous studies, we emphasized US findings of acute ileal diverticulitis as a major strength in our study. Patients with acute ileal diverticulitis often present with acute RLQ pain, which can mimic the symptoms of acute appendicitis. In emergency settings, US could be a valuable screening modality for excluding acute appendicitis and diagnosing acute ileal diverticulitis.

## 5. Conclusions

In conclusion, acute ileal diverticulitis presents as acute RLQ pain mimicking appendicitis, and it should be considered in the differential diagnosis of RLQ pain in elderly patients. Knowledge of the characteristic CT and US findings of acute ileal diverticulitis allows radiologists to diagnose it accurately, and as a result, the patients can be successfully treated with conservative management.

## Figures and Tables

**Table 1:** Clinical characteristics of the 17 patients with acute ileal diverticulitis.

No.	Sex	Age	Chief Complaint	Associated Symptoms	P.E.	B.T.	WBC	CRP	Clinical Impression	Treatment	Hospital Stay (Days)
P1 *	M	32	RLQ pain	Epigastric pain	RLQ Td	36.5	9900	17.9	Stump appendicitis	Antibiotics	9
P2 *	M	67	RLQ pain	None	RLQ Td	37.9	11,400	139	Appendicitis	Antibiotics	13
P3 *^,†^	M	56	RLQ pain	Febrile sense	RLQ Td	36.7	11,200	51.2	Appendicitis	Referral	NA
P4	M	78	RLQ pain	Diarrhea	RLQ Td	37.2	17,100	82.74	Diverticulitis	Antibiotics	6
P5 *	F	42	RLQ pain	Nausea, chills	RLQ Td	37	11,700	10.7	Appendicitis	Antibiotics	9
P6	M	60	RLQ pain	Vomiting	RLQ Td	38.2	6300	91.4	Appendicitis	Antibiotics	9
P7 *	F	70	RLQ pain	Epigastric pain	Normal	36	14,600	NA	Appendicitis	Antibiotics	12
P8	F	48	RLQ pain	Febrile sense	RLQ Td	36.6	10,300	28.07	Diverticulitis	Antibiotics	8
P9 *	F	63	RUQ and RLQ pain	Febrile sense, vomiting, diarrhea	RUQ and RLQ Td	38.3	12,100	132	Cholecystitis	Antibiotics	17
P10	M	38	RLQ pain	Febrile sense, chills, diarrhea	RLQ Td	37	NA	NA	Appendicitis	Antibiotics	9
P11	F	37	RLQ pain	Periumbilical pain	RLQ Td	37.6	23,100	163.19	Appendicitis	Antibiotics	8
P12 *	M	73	General weakness	Febrile sense, nausea, vomiting	RLQ Td	36.5	12,000	24.15	CAPD peritonitis	Antibiotics	13
P13	M	48	Fever	Chills	Normal	37.7	11,000	76	FUO	Antibiotics	6
P14 *	M	69	Dyspnea	Febrile sense, chills, abdominal pain	Normal	39.7	13,100	120	FUO	Antibiotics	13
P15	F	34	RLQ pain	None	RLQ Td	NA	15,500	NA	Appendicitis	Conservative	0
P16	M	66	RLQ pain	Febrile sense, vomiting	RLQ Td	39.8	7900	94.38	Appendicitis	Antibiotics	9
P17	M	59	RLQ pain	None	RLQ Td	NA	NA	NA	Appendicitis	Antibiotics	0

Note—No. = Number, P.E. = Physical examination, B.T. = Body temperature, WBC = White blood cell, CRP = C-reactive protein, RLQ = Right lower quadrant, Td = Tenderness, CAPD = Continuous ambulatory peritoneal dialysis, FUO = Fever of unknown origin, NA = Nonapplicable. * Perforated diverticulitis based on computed tomography or ultrasonography findings. ^†^ The patient, who was recommended to undergo surgery, was transferred to another hospital and further follow-up was lost.

**Table 2:** CT findings for acute ileal diverticulitis.

CT Findings	No. (%) of Patients
Number of ileal diverticulum	
Single	8 (47.1)
Multiple	9 (52.9)
Location	
Mesenteric	17 (100)
Antimesenteric	0 (0)
Inflamed diverticulum	
Identification	16 (94.1)
Size (mm) *	12.2 ± 3.4
Fecalith	3 (18.8)
Mesenteric fat infiltration	17 (100)
Mild	6 (35.3)
Moderate	5 (29.4)
Severe	6 (35.3)
Ileal wall thickening	17 (100)
Perforation ^†^	5 (29.4)
Venous gas	2 (11.8)
Venous thrombosis	2 (11.8)
Ileus	4 (23.5)
Normal appendix ^‡^	15 (88.2)
Other diverticula	12 (70.6)

Note.—No. (%) = Number (percentage). * Data are mean ± standard deviation. ^†^ Based on findings of abscess, extraluminal fluid with air and/or focal defect in the diverticular sac. ^‡^ The remaining 2 patients did not show normal appendix due to appendectomy (*n* = 1) and invisible appendix (*n* = 1).

**Table 3:** US findings for acute ileal diverticulitis.

US Findings	No. (%) of Patients
Inflamed diverticulum	
Identification	17 (100)
Size *	10.8 ± 3.8
Echogenicity	
Homogenous hypoechoic sac	5 (29.4)
Hypoechoic sac with internal strong echo	7 (41.2)
Central hyperechoic fecalith with peripheral hypoechoic rim	5 (29.4)
Peridiverticular inflamed fat	17 (100)
Ileal wall thickening	16 (94.1)
Perforation	7 (41.2)
Abscess	6 (35.3)
Extraluminal air bubble	6 (35.3)
Color Doppler imaging	
Increased color flow of diverticulum and surrounding fat	17 (100)

Note.—No. (%) = Number (percentage). * Data are mean ± standard deviation.

**Table 4:** Comparison of clinical features and diverticular size between perforation and non-perforation groups.

Parameters	Perforation (*n* = 7)	Non-Perforation (*n* = 9)	*p* Value
Age *	59.4 ± 14.7	52.0 ± 14.0	0.486
Sex			0.266
Male	4	6	
Female	3	3	
Fever	2 (33.3%)	5 (62.5%)	0.143
Leukocytosis	5 (83.3%)	6 (75%)	>0.05
Length of hospital stay (days) *	12.3 ± 2.5	6.1 ± 3.4	0.002
Size of inflamed diverticulum (mm) *	8.8 ± 1.6	12.7 ± 4.0	0.017

* Mann–Whitney test. Otherwise, Fisher’s exact.

## Data Availability

No new data were created or analyzed in this study. Data sharing is not applicable to this article.
